# Exploring the significance of GNG11, LPAR1, and AGTR1 in early diagnosis and prognosis of cervical cancer: A correlative analysis with clinical characteristics

**DOI:** 10.12669/pjms.41.10.12118

**Published:** 2025-10

**Authors:** Xiaojuan Wang, Fang Wang, Wenjing Yang, Yunxia Li

**Affiliations:** 1 Xiaojuan Wang, Medical Oncology, General Hospital of Ningxia Medical University Cancer Hospital, Yinchuan 750004, Ningxia, China; 2 Fang Wang, Medical Oncology, General Hospital of Ningxia Medical University Cancer Hospital, Yinchuan 750004, Ningxia, China; 3 Wenjing Yang, Medical Oncology, General Hospital of Ningxia Medical University Cancer Hospital, Yinchuan 750004, Ningxia, China; 4 Yunxia Li, Medical Oncology, General Hospital of Ningxia Medical University Cancer Hospital, Yinchuan 750004, Ningxia, China

**Keywords:** G-protein gamma subunit-11(GNG11), Cervical cancer, Early diagnosis, Prognosis, Clinical characteristics

## Abstract

**Objective:**

To investigate the significance of G-protein gamma subunit 11 (GNG11) expression in the diagnosis and prognosis of cervical cancer (CC).

**Methodology:**

This was a retrospective study. Differentially expressed genes in CC were retrieved from the Gene Expression Omnibus (GEO). A cohort of 68 CC patients from General Hospital of Ningxia Medical University Cancer Hospital was enrolled in the study from March 2021 to March 2024. Immunohistochemistry was employed to evaluate the expression levels of G-protein subunit γ 11 (GNG11), lysophospholipid receptor 1 (LPAR1), and angiotensin II receptor 1 (AGTR1) in cancerous and pericancerous tissues of CC patients. The correlations between the positive expressions of GNG11, LPAR1, or AGTR1 and the clinicopathological parameters of CC patients were analyzed.

**Results:**

A significant correlation was observed between low positive expression of GNG11 and tumor size (*P*=0.0068), FIGO stage (*P*=0.0282), and lymph node metastasis (*P*=0.0101) in CC patients. Similarly, low positive expression of LPAR1 was correlated with FIGO stage (*P*=0.0024) and lymph node metastasis (*P*=0.0203). Patients exhibiting high positive expression of GNG11 demonstrated prolonged OS (*P*=0.0001) and RFS (*P*=0.0422) compared to those with low expression. Cox regression analysis revealed that FIGO stage (*P*=0.014), lymph node metastasis (P=0.002), and GNG11 expression (P=0.026) emerged as independent prognostic indicators for OS in CC patients.

**Conclusion:**

The positive expression level of GNG11 is intricately linked to the early diagnosis and prognosis of CC, underscoring its potential as a significant diagnostic and prognostic indicator.

## INTRODUCTION

Cervical cancer (CC) remains one of the primary causes of female mortality worldwide. Research has demonstrated that CC patients remain at a substantial risk of recurrence and metastasis subsequent to combined treatment with surgery, radiotherapy, or chemotherapy.^1^,^2^ Consequently, the identification of specific biomarkers is crucial for developing new therapeutic targets and strategies, holding profound clinical significance for the enhancement of patient survival rates. The G-protein subunit γ 11 (GNG11), a constituent of the guanine nucleotide-binding protein (G protein) γ family, encodes a lipid-anchored cell membrane protein.^3^ As a member of heterotrimeric G proteins, GNG11 plays a pivotal role in the transmembrane signaling systems.^4^ GNG11 has been demonstrated to be a hub gene associated with the prognosis of ovarian cancer.^5^ Nevertheless, the expression of GNG11 and its role in the diagnosis and prognosis of CC have been scarcely reported. This study, based on various bioinformatics databases and experiments, conducted an objective analysis of the correlation between GNG11 positive expression and clinical characteristics in CC patients. The objective was to elucidate the expression patterns of GNG11 in CC, ascertain its diagnostic and prognostic value, and thereby contribute novel perspectives to the clinical management of CC.

## METHODOLOGY

This was a retrospective study. Cancerous and adjacent non-cancerous tissues were collected from CC patients admitted to General Hospital of Ningxia Medical University Cancer Hospital from March 2021 to March 2024. The study cohort comprised 68 patients, aged 28 to 70 years, with a mean age of 48.26±8.30 years. Pathological type: 50 cases of squamous cell carcinoma and 18 cases of other types; Tumor size: <3cm in 32 cases and ≥3cm in 36 cases; FIGO stage^6^: 49 cases in stages I-II and 19 cases in stages III-IV; Human papillomavirus (HPV) status: 14 negative and 54 positive cases; Lymph node metastasis: 45 cases in N0 stage and 23 cases in N1 stage or higher. Surgically excised cancerous and pericancerous normal tissues (3-5 cm from the cancerous tissues) were stored at -80°C for further analysis.

### Ethical Approval:

The study was approved by the Institutional Ethics Committee of General Hospital of Ningxia Medical University Cancer Hospital (No.: KYLL-2022-0665; Date: August 28, 2022), and written informed consent was obtained from all participants.

### Inclusion criteria:

- Patients pathologically diagnosed with CC.
- Those who had not undergone radiotherapy or chemotherapy prior to surgery.
- Those with complete clinical data.

### Exclusion criteria:

- Presence of other malignant tumors.
- Presence of immune or respiratory system diseases.
- Significant dysfunctions in the heart, liver, or kidneys.

### Instruments and Reagents:

The immunohistochemical antibodies targeting AGTR1 (PB0492), LPAR1 (BM5308), and GNG11 (A13707) were procured from Wuhan Boster Biological Technology Co., Ltd. The DAB staining kit was procured from Beijing Solarbio Bioscience & Technology Co., Ltd.

### Bioinformatics Analysis:

Data sets related to cervical cancer (CC) from the Gene Expression Omnibus (GEO), specifically GSE7410 and GSE9750, were retrieved for analysis of differentially expressed genes in CC. Criteria for selection included adj.P.Value<0.05 and |LogFoldChange|>1. Subsequently, the DAVID online platform (https://david.ncifcrf.gov) facilitated KEGG functional enrichment analysis of the identified differential genes, and the STRING online platform (https://string-db.org/) was utilized for the analysis of protein-protein interaction (PPI) networks targeting specific genes.

### Immunohistochemistry:

Preserved cancerous and pericancerous tissues were initially fixed in paraformaldehyde, followed by gradient alcohol dehydration, paraffin embedding, and sectioning. The sections were subjected to antigen retrieval using citric acid buffer solution through heating. After rinsing with PBS, primary antibodies GNG11, LPAR1, and AGTR1 were added and incubated overnight at 4°C. Staining was performed using a DAB kit, followed by hematoxylin counterstaining. After gradient alcohol dehydration, sections were mounted with neutral gum. An automated slide stainer was utilized for detection purposes. Immunohistochemical staining results were independently evaluated by a gynecologist and two pathologists using a semiquantitative scoring system to calculate positive expression rates.

### Patient Follow-up:

Patients were followed up via telephone after discharge, with the follow-up period concluding in March 2024. Overall Survival (OS) was defined as the interval from the date of diagnosis to the date of death or the end of the follow-up period. Recurrence-Free Survival (RFS) was defined as the interval from the date of diagnosis to the date of tumor recurrence or metastasis, with time measured in months.

### Statistical Analysis:

Statistical analyses were performed using SPSS 21.0 (IBM Corp., Armonk, NY, USA). Differences were compared using the t-test. The associations between the positive expressions of GNG11, LPAR1, and AGTR1 and the clinical characteristics of CC patients were analyzed using the chi-square test. Survival curves were plotted using the Kaplan-Meier method, with univariate analysis performed via Log-rank tests. Multivariate survival analysis was conducted using the Cox regression model. A P-value of <0.05 was considered to indicate a statistically significant difference.

## RESULTS

The differentially expressed genes in GSE7410 and GSE9750 are illustrated in Fig.2A and 2B, respectively. Intersections of upregulated and downregulated genes from both datasets are presented in Fig.2C and 2D respectively, revealing 169 common upregulated genes and 304 common downregulated genes. Subsequent KEGG functional enrichment analysis of these upregulated and downregulated genes revealed a significant enrichment in biological processes associated with cancer (hsa05200: Pathways in cancer), as illustrated in Fig.2E and 2F.

**Fig.1:**
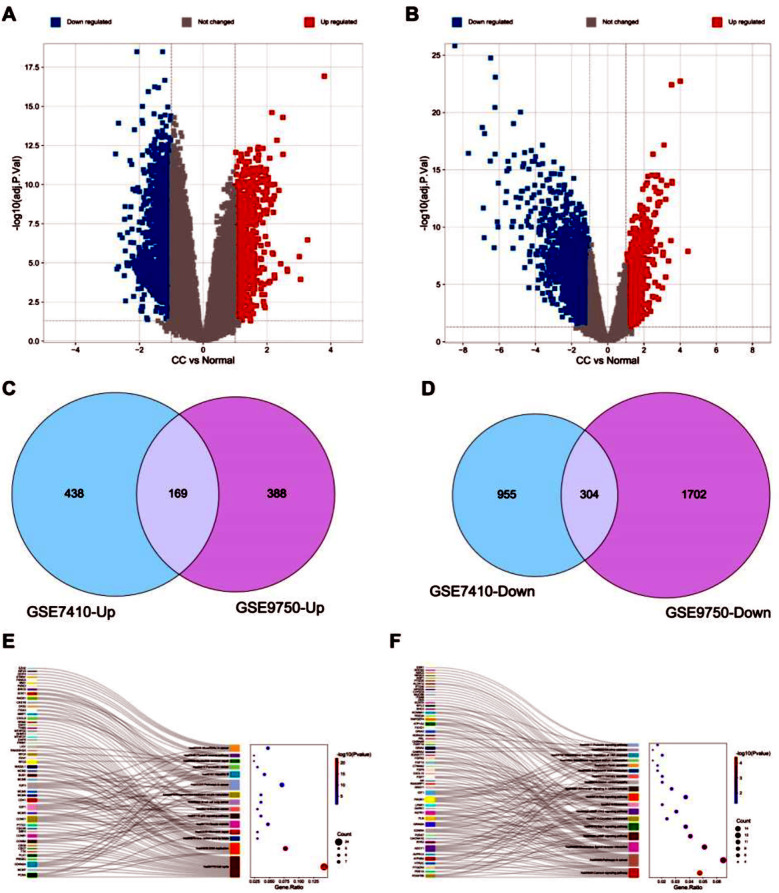
Differentially Expressed Gene Analysis Results. ***Note:*** A: Volcano plot of differentially expressed genes in GSE7410; B: Volcano plot of differentially expressed genes in GSE9750; C: Intersection of upregulated genes in GSE7410 and GSE9750; D: Intersection of downregulated genes in GSE7410 and GSE9750; E: KEGG enrichment analysis results for upregulated genes; F: KEGG enrichment analysis results for downregulated genes.

**Fig.2:**
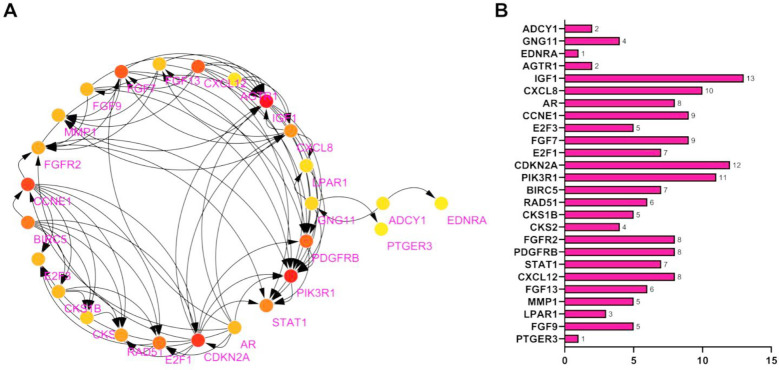
PPI Analysis Results. ***Note:*** A: PPI network analysis plot; B: Number of gene nodes.

A subset of 12 upregulated genes and 19 downregulated genes (Table-I) in CC demonstrated significant enrichment in cancer-related biological processes. These 31 genes were subjected to protein-protein interaction (PPI) analysis (Fig.2A) and further filtered using the cytohubba plugin (Fig.2B). GNG11, LPAR1, and AGTR1, which lack relevant studies in the context of CC, were included for subsequent analysis.

**Table-I:** Differentially Expressed Genes Enriched in Cancer Biological Processes.

DEGs	Gene name
Upregulated	RAD51, CXCL8, CCNE1, STAT1, CDKN2A, MMP1, ITGA3, CKS2, E2F1, BIRC5, E2F3, CKS1B
Downregulated	PDGFRB, DAPK1, PTGER3, LPAR1, ADCY1, PIK3R1, IGF1, GNG11, RASGRP1, AR, FGF7, EDNRA, CXCL12, FGF9, AGTR1, CTNNA2, FGF13, FGFR2, RUNX1T1

**Fig.3:**
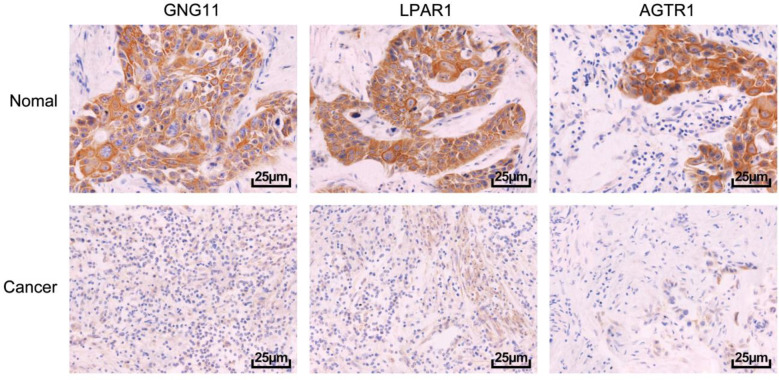
Positive Expression of GNG11, LPAR1, and AGTR1 in Cancerous and Pericancerous Tissues of CC Patients.

The expression of GNG11, LPAR1, and AGTR1 in cancerous and pericancerous tissues of CC patients was determined by immunohistochemistry. The findings revealed a marked decrease in the positive expression of GNG11, LPAR1, and AGTR1 in cancerous tissues compared to pericancerous tissues (Fig.3). Based on the median positive expression rates of GNG11, LPAR1, and AGTR1, 68 cases of CC patients were stratified into high-expression and low-expression groups, followed by statistical analysis of clinicopathological parameters. The findings (Table-II) underscored a significant association between low positive expression levels of GNG11 and tumor size (*P*=0.0068), FIGO stage (*P*=0.0282), and lymph node metastasis (*P*=0.0101) in CC patients. Similarly, low positive expression levels of LPAR1 were associated with FIGO stage (*P*=0.0024) and lymph node metastasis (*P*=0.0203). Low positive expression levels of AGTR1 were significantly associated only with FIGO stage (*P*=0.0061). Consequently, GNG11 and LPAR1 were selected for further analysis.

**Table-II:** Relationship between the Expression Levels of GNG11, LPAR1, and AGTR1 and Clinicopathological Characteristics of CC Patients.

Characteristics		n	GNG11			LPAR1			AGTR1		
		Low (n=38)	High (n=30)	P	Low (n=36)	High (n=32)	P	Low (n=31)	High (n=37)	P
Age (years)	<50	42	27	15	0.0860	20	22	0.3218	23	19	0.0794
	≥50	26	11	15		16	10		8	18	
Histological type	SCC	50	31	19	0.1051	24	26	0.2707	25	25	0.2764
	Non-SCC	18	7	11		12	6		6	12	
Tumor size (cm)	<3	32	12	20	0.0068	14	18	0.2236	13	19	0.4736
	≥3	36	26	10		22	14		18	18	
FIGO stage	Ⅰ-Ⅱ	49	23	26	0.0282	20	29	0.0024	17	32	0.0061
	Ⅲ-Ⅳ	19	15	4		16	3		14	5	
HPV virus	Negative	14	9	5	0.5559	8	6	0.7720	5	9	0.5496
	Positive	54	29	25		28	26		26	28	
Lymph node metastasis	N0	45	20	25	0.0101	19	26	0.0203	24	21	0.1217
	N1 or above	23	18	5		17	6		7	16	

**Fig.4:**
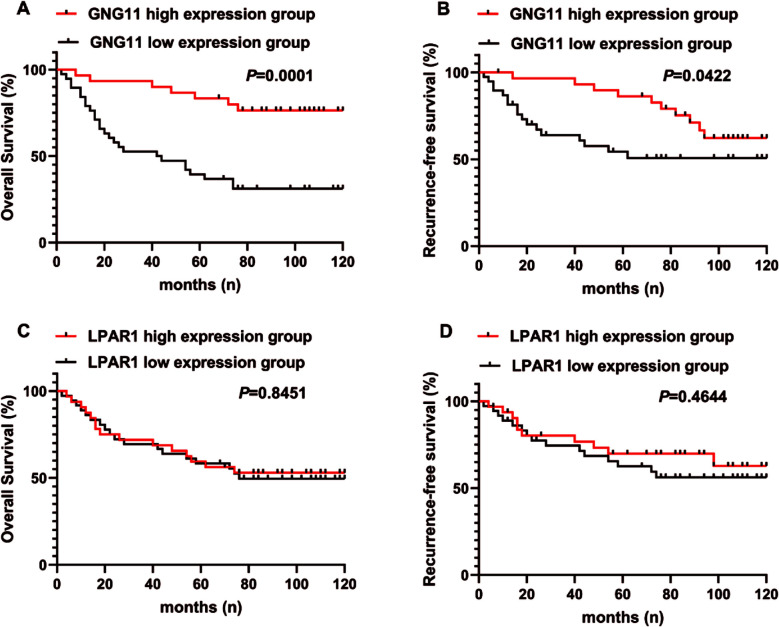
Positive Correlation between GNG11 Positive Expression and Overall Survival as well as RFS in CC Patients. ***Note:*** A-B: OS and RFS analysis results of CC patients with high and low GNG11 positive expression; C-D: OS and RFS analysis results of CC patients with high and low LPAR1 positive expression.

Survival analysis of CC patients was undertaken based on the positive expression of GNG11 and LPAR1. As illustrated in Fig.4, the survival time of the GNG11 high positive expression group was significantly longer than that of the low positive expression group for both OS (*P*=0.001, Fig.4A) and RFS (*P*=0.0422, Fig.4B). No statistically significant differences were observed in the positive expression of LPAR1 with regard to OS and RFS in CC patients (Fig.4C and 4D).

Univariate analysis identified tumor size (*P*<0.0001), FIGO stage (*P*=0.0193), lymph node metastasis (*P*=0.0087), and GNG11 positive expression (*P*=0.0001) as prognostic factors influencing OS (Table-III). Conversely, age, histological type, and HPV were not associated with OS in CC patients. Multivariate Cox analysis revealed that FIGO stage (*P*=0.014), lymph node metastasis (*P*=0.002), and GNG11 positive expression (*P*=0.026) were independent prognostic factors for OS (Table-IV).

**Table-III:** Univariate Survival Analysis Results for OS in CC Patients.

Variables	OR	95%CI	P
Age (<50, ≥50)	1.282	0.6313-2.605	0.4751
Histological type (SCC, Non-SCC)	0.8526	0.3964-1.834	0.6927
Tumor size (<3, ≥3)	4.796	2.410-9.542	<0.0001
FIGO stage (Ⅰ-Ⅱ, Ⅲ-Ⅳ)	2.199	1.077-4.491	0.0193
HPV virus (Negative, Positive)	0.9876	0.4274-2.282	0.9765
Lymph node metastasis (N0, N1 or above)	2.425	1.191-4.939	0.0087
GNG11 positive expression (high, low)	4.391	2.214-8.708	0.0001

**Table-IV:** Multivariate Cox Analysis Results for OS in CC Patients.

Variables	OR	95%CI	P
Tumor size (<3, ≥3)	2.088	0.785-5.550	0.114
FIGO stage (Ⅰ-Ⅱ, Ⅲ-Ⅳ)	3.181	1.264-8.005	0.014
Lymph node metastasis (N0, N1 or above)	3.722	1.609-8.611	0.002
GNG11 positive expression (high, low)	3.022	1.176-7.763	0.026

## DISCUSSION

This study demonstrates that the positive expression of GNG11 is markedly suppressed in CC, and a low level of GNG11 expression is significantly associated with poor prognosis, thereby emerging as a potential biomarker for evaluating postoperative overall survival (OS) and recurrence-free survival (RFS) in patients. Cervical cancer (CC), the most prevalent gynecological malignancy, has exhibited a trend of younger onset ages in recent years, posing a severe threat to women’s life and health.^7^-^9^ In this study, CC-related datasets were screened from the GEO database, and differential gene expression analysis was conducted to identify candidate targets for CC. KEGG and PPI analyses revealed that GNG11, LPAR1, and AGTR1, as key factors in cancer biological processes, are significantly downregulated in CC, suggesting their potential role as tumor suppressor genes, which is of profound importance in unveiling the pathogenesis of CC. Previous studies on GNG11 have predominantly been confined to lung cancer-related research. It has been observed that GNG11 expression is downregulated in lung cancer, and this downregulation is associated with poor OS in never-smoking female patients.^10^-^12^ GNG11 has also been identified as a core gene associated with triple-negative breast cancer, exhibiting downregulated expression in tumor tissues.^13^,^14^ Furthermore, GNG11 may play a crucial role in the treatment of colorectal cancer.^15^ Additionally, GNG11 has been highlighted as the most valuable protein with downregulated expression in CC.^16^ The immunohistochemistry results were consistent with the trends observed in the GEO database, demonstrating that the positive expression rates of GNG11, LPAR1, and AGTR1 in cancerous tissues of CC patients are substantially lower than in pericancerous normal tissues.

The study further explored the correlation between positive GNG11 expression and clinical characteristics of CC, revealing that low positive expression of GNG11 is associated with adverse features such as tumor size, FIGO stage, and lymph node metastasis, but not with age, histological type, or HPV infection. LPAR1 has previously been validated as a potential target in melanoma^17^ and osteosarcoma^18^ treatments; however, its role in CC remains unexplored. This study not only confirmed the downregulation of LPAR1 in CC but also demonstrated that low positive expression of LPAR1 is associated with FIGO stage and lymph node metastasis. AGTR1 has been shown to be upregulated in ovarian cancer^19^; nevertheless, other studies have identified its reduced expression in lung adenocarcinoma, where overexpression of AGTR1 can suppress progression via the PI3K/AKT pathway.^20^ Although AGTR1 is confirmed to be downregulated in CC in this study, it is found to be correlated only with FIGO stage when analyzing its association with clinical parameters. Collectively, these findings suggest a significant association between LPAR1 positive expression and LPAR1 as well as CC progression.

Upon following up with patients and recording survival data, the findings demonstrate that patients exhibiting high GNG11 expression experience superior prognostic outcomes, extended survival periods, and enhanced OS and RFS. This suggests that maintaining higher levels of positive GNG11 expression may potentially ameliorate clinical outcomes for CC patients. Conversely, no statistically significant association is observed between LPAR1 positive expression and OS or RFS in CC patients. Further analysis of prognostic factors affecting OS in CC patients reveals that tumor size, FIGO stage, lymph node metastasis, and the positive expression level of GNG11 are significantly related to OS, whereas age, histological type, and HPV infection are not linked to patient survival. Furthermore, multivariate Cox analysis indicates that tumor size, lymph node metastasis, and GNG11 positive expression level maintain independent correlations with OS in CC patients.

### Limitations:

It includes a small sample size. More indexes of pain behaviors and inflammatory mediators should be investigated. In view of this, more samples should be included in future studies to further validate the findings of this study.

## CONCLUSIONS

Low positive expression of GNG11 indicates a poor prognosis for CC patients. GNG11 positive expression could serve as a crucial indicator for the early diagnosis and prognostic prediction of CC, thereby contributing valuable data for its clinical treatment.

### Authors’ Contributions:

**XW:** Conceived and designed the study. Literature search,

**FW:** Collected the data and performed the analysis. Critical review.

**WY** and **YL:** Was involved in the writing of the manuscript and is responsible for the integrity of the study. All authors read and approved the final manuscript.
